# Reduced infarct size in neuroglobin-null mice after experimental stroke *in vivo*

**DOI:** 10.1186/2040-7378-4-15

**Published:** 2012-08-20

**Authors:** Zindy Raida, Christian Ansgar Hundahl, Jesper Kelsen, Jens Randel Nyengaard, Anders Hay-Schmidt

**Affiliations:** 1 Department of Neuroscience and Pharmacology, Faculty of Health Sciences, University of Copenhagen, Copenhagen, Denmark; 2 Department of Physiology, University of Tartu, Tartu, Estonia; 3 Centre of Excellence for Translational Medicine, University of Tartu, Tartu, Estonia; 4 Department of Clinical Biochemistry, University Hospital Bispebjerg, Copenhagen, Denmark; 5 Department of Neurosurgery, University Hospital Copenhagen (Rigshospitalet), Copenhagen, Denmark; 6 Stereology and Electron Microscopy Research Laboratory, Centre for Stochastic Geometry and Advanced Bioimaging, Aarhus University, Aarhus, Denmark; 7 The Panum Institute; Department of Neuroscience and Pharmacology, University of Copenhagen, Blegdamsvej 3, 2200, Copenhagen N, Denmark

**Keywords:** Neuroglobin, Knock out, Ischemia, Immunohistochemistry, Brain

## Abstract

**Background:**

Neuroglobin is considered to be a novel important pharmacological target in combating stroke and neurodegenerative disorders, although the mechanism by which this protection is accomplished remains an enigma. We hypothesized that if neuroglobin is directly involved in neuroprotection, then permanent cerebral ischemia would lead to larger infarct volumes in neuroglobin-null mice than in wild-type mice.

**Methods:**

Using neuroglobin-null mice, we estimated the infarct volume 24 hours after permanent middle cerebral artery occlusion using Cavalieri’s Principle, and compared the infarct volume in neuroglobin-null and wild-type mice. Neuroglobin antibody staining was used to examine neuroglobin expression in the infarct area of wild-type mice.

**Results:**

Infarct volumes 24 hours after permanent middle cerebral artery occlusion were significantly smaller in neuroglobin-null mice than in wild-types (p < 0.01). Neuroglobin immunostaining of the penumbra area revealed no visible up-regulation of neuroglobin protein in ischemic wild-type mice when compared to uninjured wild-type mice. In uninjured wild-type mice, neuroglobin protein was seen throughout cortical layer II and sparsely in layer V. In contrast, no neuroglobin-immunoreactive neurons were observed in the aforementioned layers of the ischemia injured cortical area, or in the surrounding penumbra of ischemic wild-type mice. This suggests no selective sparing of neuroglobin expressing neurons in ischemia.

**Conclusions:**

Neuroglobin-deficiency resulted in reduced tissue infarction, suggesting that, at least at endogenous expression levels, neuroglobin in itself is non-protective against ischemic injury.

## Background

Neurons are particularly sensitive to hypoxia- and ischemia-related stress due to the very high metabolic demand of the brain [1] and were believed to lack an endogenous oxygen storage/diffusion system. In 2000 Burmester and co-workers discovered a neuronal specific heme-globin, Neuroglobin (Ngb), in the mouse brain [2]. Due to structural similarity with hemoglobin and myoglobin, and ability to bind oxygen, Ngb was suggested to function as global oxygen provider or reactive oxygen species (ROS) scavenger in the brain [3-6]. Ngb was therefore proposed to be a novel pharmacological target in combating neurodegenerative disorders [7] (for review see Dietz 2011 [8]). A number of *in vitro* and *in vivo* studies have addressed the role of Ngb in neuroprotection and suggest that Ngb can protect against hypoxic/ischemic neuronal damage, although the mechanism of protection remains enigmatic. Increased Ngb immunoreactivity in the ischemic hemisphere compared to the non-ischemic hemisphere has been reported following transient occlusion of the middle cerebral artery (tMCAo) [9]. Also, Ngb over-expressing mice are reported to have smaller infarct volumes and reduced oxidative stress markers in the brain after transient focal [10] or global [11] ischemia whereas down-regulation of Ngb worsens the ischemic outcome [12]. In a recent paper we probed the question of possible Ngb neuronal protection after severe long-term hypoxia using genetically Ngb-deficient (Ngb-null) mice. We detected no effect of Ngb-deficiency on neuronal survival after chronic hypoxia, nor did we find any effect on animal survival rate. Instead, Ngb deficiency appears to up-regulate a few core hypoxia response genes [13]. Thus, despite numerous studies on the relation of Ngb and hypoxia/ischemia there remains a lack of consensus as to the role of Ngb in neuroprotection. In the current study we used a permanent middle cerebral artery occlusion (pMCAo) model [14,15] on Ngb-null mice to determine whether Ngb deficiency exacerbates the damage seen after brain ischemia. Infarct volume 24 h after pMCAo was estimated using Cavalieri’s principle. Specific antibody staining was used to examine Ngb expression in the infarct area in wild-type (WT) mice. To our knowledge, this is the first study to examine the post-ischemic response in Ngb-null mice.

## Methods

Animal care and all experimental procedures were conducted in accordance with Danish Ministry of Justice. The Danish National Committee for Ethics in Animal Research approved the experimental protocol in accordance with the European Community Council’s Directive of November 24^th^ 1986 (86/609/EEC).

Animals were housed at the animal facility center, at the Panum Institute, University of Copenhagen in a 12:12 h light: dark cycle (lights on at 6 a.m.; lights off at 6 p.m.). Daily routines were carried out between 7 a.m. and 4 p.m. by authorized personnel. Standard laboratory chow and water were provided ad libitum, as well as soaked standard laboratory chow and nutritional gel postoperative.

### Study design

Male C57BL/6 (8 weeks old) were randomized to one of the following three groups: I. WT pMCAo (n = 25), II. Ngb-null pMCAo (n = 14), III. Uninjured-WT (n = 23). All animals were euthanized after 24 hours. WT pMCAo (n = 12), Ngb-null pMCAo (n = 13) and uninjured-WT (n = 10) were imbedded in paraffin and sectioned for infarct volume estimation whereas WT pMCAo (n = 6), Ngb-null pMCAo (n = 1) and uninjured-WT (n = 6) were frozen sectioned in 40 μm coronal slices in replicas of four and used for immunohistochemistry (IHC). For western blotting, uninjured WT (n = 7) and WT pMCAo (n = 7) were used.

### Animal preparation

#### Ngb deficient mice

Development of a Ngb-null mouse model was performed by GenOway (Lyon, France) and described in detail in Hundahl et al 2011 [13]. In brief, using the Cre-LoxP method exon 2–3 of the Ngb gene was removed, and transmitted through germ-line resulting in embryonic Ngb deficiency. Ngb-null mice were backcrossed to C57BL/6J genomic background for 9 generations.

### Anesthesia

Anesthesia was induced by inhalation of 8% sevoflurane (Abbott Laboratories, Inc) delivered in a mixture of medical grade air (content as atmospheric air) in an anesthetic chamber. The animal was placed on a plastic support to fixate the animal during intubation. General anesthesia was maintained with a mixture of Sevoflurane 5% and medical grade air by an open breathing circuit connected to the support via a 1 mL syringe, which was used as a facemask. Once the animal was fixated a fiber optic arm of a strong halogen light source was positioned over the neck of the animal to light up the trachea from the outside. The animals were orally intubated using a cannulae for mice (OD 1,2 mm, L 30 mm, Hugo-Sachs Elektronik Harvard Apparatus GmbH, Germany) and connected to a mouse ventilator (Minivent type 845 Hugo-Sachs Elektronik Harvard Apparatus GmbH, Germany) and a Capnograph (Type 340 Hugo-Sachs Elektronik Harvard Apparatus GmbH, Germany). Anesthesia was maintained with a mixture of 5.5% sevoflurane and medical air (delivered by Aga, Denmark) at a rate of 120-130 breaths/min and an inspiratory volume of 200 μL. To secure normal physiological tissue oxygenation ventilator parameters were determined by a pilot experiment with periodic measurements of arterial blood gases and caphnograph measurements of the end tidal CO_2_ (ETCO_2_)_._ By making a standard curve that correlates arterial partial CO_2_ pressure with the ETCO_2_ the ventilator was adjusted exactly to fit the individual animal in order to maintain blood gases within the normal range. The whole intubation procedure was achieved in less than 3 min.

### Monitoring of physiological parameters

The core body temperature was kept at 37°C (36.86°C −37.16°C see Table 1) using a feed-back system with a temperature-controlled heating pad coupled to a rectal probe (Small homeothermic Blanket Control Unit, Hugo-Sachs Elektronik Harvard Apparatus GmbH, Germany). Controlling animal body temperature in a normal range is necessary to eliminate hypothermia which reduces [16] and hyperthermia which exacerbates [17-19] ischemic brain injury. Controlling ventilation and blood gas values is essential since hyperoxia has been demonstrated to be neuroprotective during ischemia and reperfusion [20]. Similarly, as carbon dioxide is a potent cerebral vasodilator and causes increased cerebral blood flow [21,22] hypercarbia may have a protective effect during ischemia and reperfusion [23]. Systolic blood pressure and heart rate was measured none-invasively by use of a tail cuff (Panlab Basic Unit for Indirect BloodPressure LE5001 Hugo-Sachs Elektronik Harvard Apparatus GmbH, Germany), in 14 of the animals taken by random from all three groups. This was done to visualize blood pressure fluctuations that affect regional cerebral blood flow and hence stroke outcome [24,25]. Blood pressure is also a sensitive indicator for assessing anesthetic depth [26]. A Powerlab 8/30 with LabChart Pro (ADInstruments, Oxford, United Kingdom) data acquisition system was used to collect physiological data during experiments. The experimental set-up is in accordance with The Stroke Therapy Academic Industry Roundtable [27] (STAIR) which was established in order to address the challenges encountered in finding an effective neuroprotective therapy for stroke [28-30]. All of the above mentioned factors have been reported to influence stroke model outcome tremendously [27] and is unfortunately not reported in all studies where Ngb is postulated to be neuroprotective. Physiological parameters measured are shown in Table 1.

**Table 1:** Physiological parameters

**Group**	**PMCAo WT (n = 24)**	**PMCAo Ngb-null (n = 14)**	**PMCAo-sham WT (n = 16)**
Mean systolic BP (mmHg)	115.8 ± 11.23 (n = 6)	122.1 ± 8.61 (n = 6)	100.9 ± 0.700 (n = 2)
Heart Rate (BPM)	458.5 ± 9.10 (n = 9)	425.6 ± 13.87 (n = 8)	483.3 ± 13.63 (n = 5)
Mean pCO_2_ in the exhaust air (kPa)	37.57 ± 0.59 (n = 24)	36.89 ± 1.57 (n = 14)	40.13 ± 0.36 (n = 16)
Rectal Temp. (°C)	37.09 ± 0.07 (n = 24)	37.07 ± 0.09 (n = 14)	36.96 ± 0.10 (n = 16)
Body weight preoperative (gram)	25.38 ± 0.75 (n = 24)	25.81 ± 1.48 (n = 14)	27.28 ± 1.67 (n = 16)
Body weight, day of sacrifice (gram)	22.32 ± 0.65 (n = 24)	22.64 ± 1.29 (n = 14)	24.49 ± 1.56 (n = 16)

Physiological parameters monitored during the operational procedure in all three groups. Mean values ± SE. Mann-Whitney analysis was used for the comparison between the groups.

### Behavioral test

The following tests were carried out in our pilot investigations:

1. Modified Bederson neurological scoring is a widely used test originally developed by Bederson et al 1986 [15]. We attempted to measure neurological deficits according to the following scoring system:

0 = No deficit; 1 = Flexion of contralateral torso and forelimb upon lifting the whole animal by the tail; 2 = Circling to the contralateral side, when held by the tail with the feet on the floor; 3 = Spontaneous circling to contralateral side; 4 = no spontaneous motor activity.

2. The accelerating Rotarod was used to test balance and coordination. Each animal was trained in four consecutive trials per day at day four and three days before surgery to test their motoric baseline. Their motoric abilities were tested 24 hours post surgery and compared to their individual baseline.

3. Paw reaching – the staircase test was used to test grasping abilities. Briefly animals are placed in commercially available staircase cages. Each step contains food pellets for the animals to grasp. The number of steps from which pellets have been removed provides an index of how far the mouse can reach, and the number of pellets remaining at the end of the test indicates the mouse’ success in grasping and retrieving the pellets [31].

4. Adhesive removal test [32] was used in attempt to evaluate both somatosensory and motor function. Briefly, this test consists of placing adhesive tape strips on both sides of the forelimbs. Measuring the time needed to sense and to remove the adhesives assesses animal performance.

### Surgical procedure

Before surgery all animals received an intramuscular injection of 5 μg/100 g bodyweight of atropine (Atropinsulfat 1 mg/ml, Denmark) to reduce mucus production. Bupivacain (Bupivacain SAD 5 mg/ml, Denmark) and Lidocaine (SAD 5 mg/ml) mixed 1:1 was injected subcutaneously at the incision sites to ease postoperative pain.

A skin incision between the lateral part of the orbit and the external auditory meatus was made. A burr-hole was drilled directly over the distal part of the MCA, the dura mater was removed, and the MCA was coagulated by applying bipolar forceps coupled to an electrosurgical unit (ERBE VIO 100 C Mediplast NCNielsen A/S, Denmark) [14]. The animals received a subcutaneous injection of 1.5 ml saline 37°C to prevent postoperative dehydration, and recovered in heated cages. Sham operated animals were subjected to the exact same procedure except coagulation of the MCA.

### Perfusion fixation and tissue handling

Perfusion fixation was conducted as previously described [33].Brains used for infarct volume estimation were paraffin embedded and cut into 40 μm thick coronal sections on a MICROM HM 355 (Brock and Mikkelsen A/S, Denmark). Each brain was sectioned from the forebrain to the hindbrain in series of 15. The first four sections were sampled in four series whereas section number 5-15 was discarded. Section number 16–19 was sampled in the four series and section number 20–30 was again discarded and so on. In this manner, the section distance between the slides in one sampled series were 600 μm. Sections in one series were stained with Mayer’s haematoxylin (Mayer from Th. Geyer Denmark ApS), serially mounted on glass slides, dehydrated, defatted and cover slipped. Variations in haematoxylin staining were minimized by processing sets of sections at the same time including the reference specimens, by the same technician. All tissue analyses were performed double blinded on coded specimens. Since Ngb is postulated to be up-regulated in the penumbra area we chose to stain with Mayer’s haematoxylin due to the high sensitivity and the fact that ischemia-induced neuronal morphological changes can be detected shortly after MCA occlusion. Furthermore, the level of information that can be drawn from this staining is much higher than the information given with a TTC (2,3,5-triphenyltetrazolium chloride) staining.

Three days prior to cryosectioning brains used for IHC were exchanged to a 30% sucrose PBS solution. The left non-ischemic hemispheres were marked with a small transverse incision. The forebrains were separated from the cerebellum and cut in two equally sized pieces both mounted in the coronal plane with Tissue-Tek® (Sakura Finetek Europe, Netherlands) and cut into 40 μm thick sections on a Shandon AS 200 cryostat (Anglia Scientific Instruments LTD, Cambridge England). Each brain was sectioned from the forebrain to the hindbrain in series of 4. The sections were stored in PBS with 0.1% sodium azide at 4°C.

### Western Blotting

Brains from seven sham and seven male pMCAo mice were removed 24 after the operation. The brains were divided in the two hemispheres and snap frozen on dry ice and stored at −80. Protein from the pMCAo injured or sham hemisphere was extracted using the PARIS kit (code# MA1921, Invitrogen, Carlsbad, CA, USA) supplemented with 1% Halt Phosphatase Inhibitor Cocktail (Pierce, Rockford, IL, USA) and protease inhibitors (code# P8340, Sigma Aldrich, Broendby, Denmark) according to manufacturer’s instructions.

Electrophoresis, transfer of proteins and western blotting were performed as described in [13]. Immunoblotting was conducted over night at 4°C with polyclonal guinea pig anti-Ngb (code# G, developed in house [33], diluted 1:1000) and rabbit anti-beta-actin (code# 4790, Cell Signaling, Danvers, MA, USA diluted 1:5000) as loading control. Immunoreactivity was detected with donkey anti-guinea pig (code# 706-036-148, Jackson Immunoresearch Laboratories, Baltimore, PA, USA, diluted 1:2500) and swine anti-rabbit IgG (code# P0399, Dako, Glostrup, Denmark, diluted 1:2500) horseradish peroxidase-conjugated secondary antibodies. Protein bands were visualized with enhanced chemiluminescence according to manufacturers protocol (Western Lightning Plus-ECL, PerkinElmer, Waltham, MA, USA).

### Stereology

The infarct volumes were estimated using the Cavalieri principle. This principle states that the volume of any arbitrarily shaped object may be estimated by sectioning it into a set of parallel planes with known spacing and measuring the cross sectional area *a*_*i*_ of the object in each of the *i* planes. The estimate of volume V is:

(1) $$V=t\overset{m}{\Sigma }a_{i}$$

where *t* is the known distance between sections and *m* is the number of slices into which the object is sectioned. The correct use of the Cavalieri principle for volume estimation requires that: (1) all sections of the object of interest are parallel; (2) the distance between the sections is known; and (3) the position of the first coronal slice hitting the structure of interest is random. An Olympus MVX10 MacroView microscope equipped with an Olympus DP71 Digital camera was connected to a PC. The cross sectional areas were estimated unbiased with a 2D nucleator [34] on haematoxylin stained coronal brain sections using NewCAST software (Visiopharm A/S, Hørsholm, Denmark) [34,35].

### Immunohistochemistry

IHC was performed according to previously described protocols [33]. In brief, Ngb was detected with an in-house made polyclonal rabbit antibody (code# 4836/5, in 1:100.000 dilution) against purified recombinant mouse Ngb protein [36]. The primary antibody was visualized by a (Fab)_2_ fragment of a donkey anti-rabbit antibody conjugated to biotin (Jackson Immunoresearch Laboratories, Baltimore, PA, USA, code# 706-066-152, in 1:2000 dilution) in combination with Avidin-Biotin-peroxidase Complex (ABC) (Vector labs, Burlingame, CA, USA), followed by 0.05% diaminobenzidine (DAB).

### Antibody validation

IHC was performed as described above using the following primary antibodies: Ngb was detected with either an in-house made polyclonal rabbit antibody (code# 4836/5, in 1:100.000 dilution), polyclonal rabbit antibody against amino acid 55–70 of human Ngb (code# N7162, Sigma-Aldrich, in 1:500 dilution) or polyclonal goat antibody against the an internal region of human Ngb (code# sc-22001, Santa Cruz Biotechnology, in 1:500 dilution). The primary antibodies were visualized by a (Fab)_2_ fragment of a donkey anti-rabbit antibody or goat conjugated to biotin (Jackson Immunoresearch Laboratories, Baltimore, PA, USA, code# 706-066-152 or 705-066-147, in 1:2000 dilution) in combination with ABC (Vector labs, Burlingame, CA, USA), followed by 0.05% DAB. As negative control the primary antibodies were omitted, which eliminated all staining from the respective secondary antibodies.

### Statistics

Using 6-9 mice, the experiment necessitated an effect of the treatment of roughly one standard deviation in the outcome to obtain a power of 80% to detect a statistically significant difference. We believe that this suggests that the study was sufficiently powered for detecting even modest effects of treatment on the outcome.

The significant results suggest that the power was sufficient, whereas insignificant results might have been attributed to either insufficient power or to a lack of effect of the treatment on the outcome. We thus believe that given the fact that the experiment produced highly significant results; any further post hoc analyses of power are unwarranted.

All data were analyzed using GraphPad Prism software. Infarct volumes were tested with a Mann -Whitney two-tailed test and p < 0. 05 was considered statistically significant.

## Results

### Drop outs

Three WT mice and four null mice died under surgery or did not fully recover after anesthesia. Two WT were damaged during handling. One WT was excluded from the study due to abnormal MCA anatomy with two branches running in parallel. Therefore we ended up with WT pMCAo (n = 6), Ngb-null pMCAo (n = 9) and uninjured-WT (n = 10) for paraffin embedding and infarct volume estimation.

### Physiological parameters

Of the physiological parameters recorded during surgery (Table 1) only anesthesia time was significantly different (longer in Ngb-null mice) between the genotypes. Although the animals were operated in randomized order the majority of Ngb-null operations took place in the beginning of the experiment. Mean pCO_2_ levels in the exhaust air were significantly higher in the uninjured WT when compared to the pMCAo WT.

### Behavioral test

1. Bederson test. Unfortunately the animals were too aggressive to evaluate since the animals rotated all of their body to reach and bite the investigator when handled.

2. The accelerating Rotarod. In our rotarod experiments animals performed better on the rod post surgery. We believe this was due to the fact that before surgery they did not concentrate but were jumping around on the rod smelling their neighbor and turning around on the rod with high risk of losing balance. After surgery they were much more calm and concentrated. Maybe therefore they performed better. We concluded that the test was inconclusive.

3. Paw reaching – the staircase test. This test often requires food restriction, which is never recommended in mice unless studying food deprivation. Without food deprivation our animals did not want to eats the pellets when placed in the apparatus. Also, they were very aggressive when put in the plexiglas container. The test could not be properly conducted.

4. Adhesive removal test: During the 5 recommended 5 day training period (1 trial per day) the mice became more and more aggressive. At the end it was impossible to fixate them enough to place the adhesive tape correctly and with the same amount of pressure on the paws. Therefore the test could not be properly conducted.

None of the above mentioned behavioral tests could be successfully carried out in our hands. They all turned out inconclusive or could not be carried out do to the animals becoming very aggressive when handled and trained.

### Expression of Ngb after pMCAo

**Figure 1:**
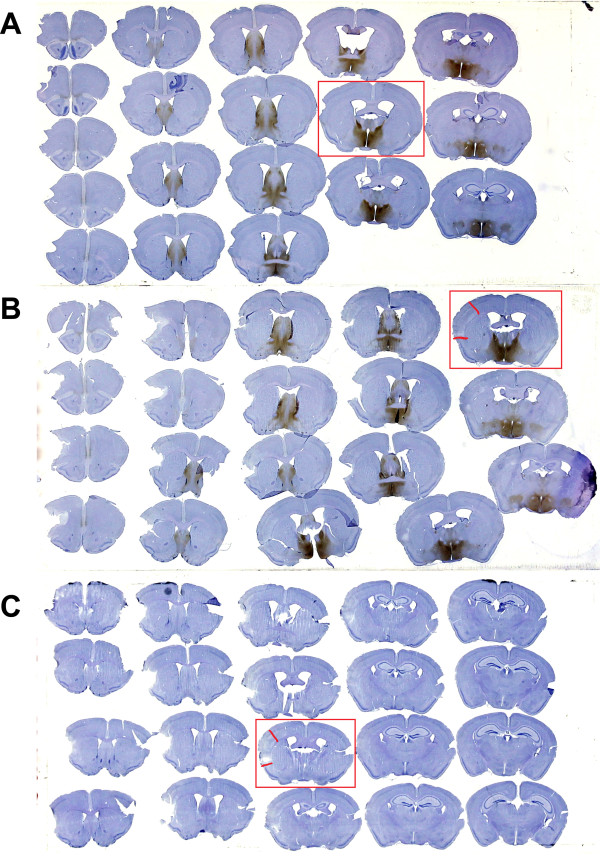
**Neuroglobin (Ngb) expression and infarct distribution.** Ngb expression (brown) and infarct distribution on Haematoxylin counter stained sections 24 hours after permanent middle cerebral artery occlusion (pMCAo). **A.** Uninjured, **B.** wild-type (WT) pMCAo and **C.** Ngb-null pMCAo. Note that there is no Ngb staining in Ngb-null mice, and that staining in cortex is very limited. The infarct is marked with red lines on the sections within red squares, which are shown in higher magnification in Figure 2.

**Figure 2:**
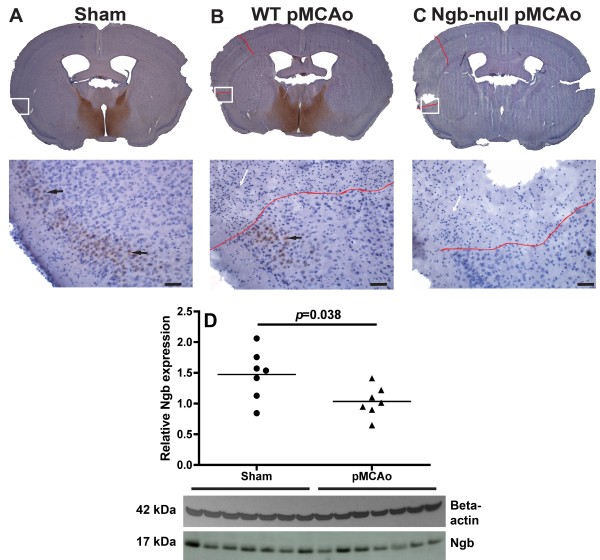
**Ngb expression in the ischemic penumbra.** Panel **A-C** shows representative sections of Ngb expression (see arrows) and infarct distributions delineated by red lines in uninjured (sham), WT and Ngb-null pMCAo mice respectively. The bottom part of the panel shows high magnification images of the area in the white squares where black arrows point at Ngb expressing neurons and white at necrotic neurons. The areas above the red lines are tissue undergoing liquefactive necrosis identified by pyknosis, karyolysis, karyohexis and rapid tissue dissolution consistent with necrotic ischemic brain injury. Note how no increase in Ngb expression is seen within or adjacent to the penumbra area (**Panel A-B** bottom) in WT pMCAo compared to sham. Also please note how no Ngb staining is seen in the infarcted area (**Panel B** bottom) suggesting no selective sparing of Ngb expressing neurons. In **D** a western blot quantification of Ngb expression in sham (SE ± 0.15) and pMCAo WT (SE ± 0.092) mice relative to beta-actin is shown. A significant (p = 0.038) down regulation of Ngb was observed in the pMCAo group compared to the sham operated group. Lines indicate mean values. Scale bar 150 μm.

Overall, Ngb immunoreactivity was observed primarily in the hypothalamus (Figure 1) and it was localized intracellularly overlapping with haematoxylin staining (see arrows in Figure 2A-B). The Ngb expression pattern and sub-cellular localization was consistent with the previous report [33]. Section cut through the area of infarction 24 hours after ischemic brain injury clearly showed liquefactive necrosis (Figure 2 panel B, C), a consistent finding in cerebral ischemia [37]. IHC studies of WT brains showed sparse Ngb expression in the cerebral cortex of both ischemic and uninjured animals (Figures 2 and 1). Visual inspection of the penumbra area revealed no up-regulation of Ngb protein in the ischemic WT when compared to uninjured WT. In uninjured WT mice, Ngb protein was seen throughout cortical layer II and sparsely in layer V. In contrast, no Ngb immunoreactive neurons were seen in the aforementioned layers of the ischemia injured cortical area or in the surrounding penumbra of WT mice. This suggests no selective sparing of Ngb expressing neurons (Figure 2 panel B bottom). No Ngb immunoreactivity was detected in ischemic Ngb-null mice (Figure 2 panel C). Western blot analysis of sham and pMCAo hemispheres from WT mice showed significantly less Ngb protein in the pMCAo operated mice (Figure 2D)

### Infarct volume

**Figure 3:**
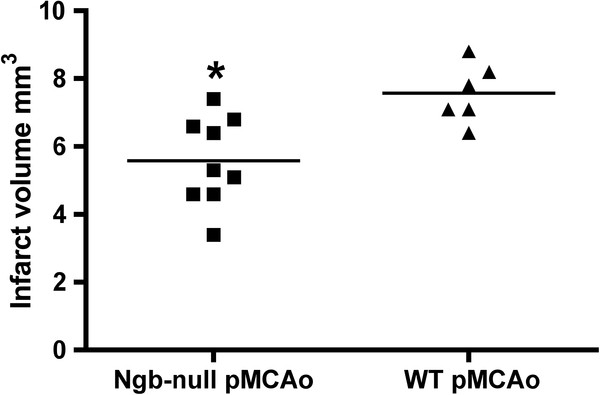
**Infarct volumes 24 hours after permanent middle cerebral artery occlusion.** Infarct volumes 24 hours after pMCAo measured with 2D nucleator and Cavalieri’s principle. Infarct volume in cortex was significantly larger in WT mice (n = 6, 7.6 mm³ SE ± 0.35 mm³) compared to matching Ngb-null (n = 9 5.7 mm³ SE ± 0.41 mm³) littermates. p < 0. 0076. Lines indicate mean values and * denote significance.

The estimated total mean infarct volumes using the 2D nucleator and the Cavalieri’s principle on Mayer’s haematoxylin stained sections 24 hours after pMCAo differed significantly between WT (7.6 mm³ SE ± 0.35 mm³) and Ngb-null mice (5.7 mm³ SE ± 0.41 mm³) (p < 0.0076 Mann-Whitney test) (Figure 3). All 25 animals in both the WT and Ngb-null group had ischemic damage within the right hemisphere 24 hours after pMCAo. In all animals permanent middle cerebral artery infarction only affected lateral aspects of the right cortical areas except in one animal, which presented injury in the cortex as well as in the underlying striatal structures. Representative infarct distributions are shown in Figures 2 and 1. No brain damage was observed among uninjured animals, data not shown.

### Antibody validation

**Figure 4:**
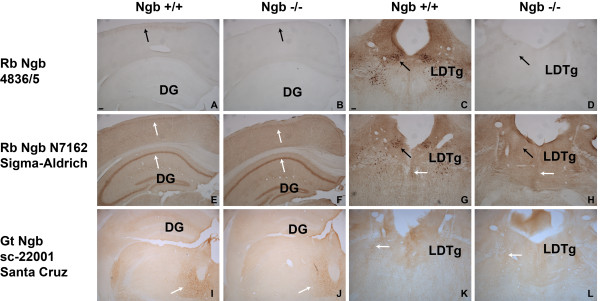
**Immunohistochemical staining for Ngb in the brain.** Immunohistochemical staining for Ngb in the brain of Ngb +/+ and Ngb -/- mice. In house made rabbit antibody 4836/5 showed specific (Ngb) staining (black arrow, signifying specific staining) in Ngb +/+ mice (**A, C)**, which was abolished in Ngb -/- mice **(B, D)**. Sigma rabbit Ngb antibody N7162 stained most neurons of cortex, hippocampus (white arrows, signifying unspecific staining) and neurons of the laterodorsal tegmental nucleus (LDTg) (black arrow) **(E, G)**. Only the staining in the large LDTg neurons was abolished in Ngb -/- mice **(F, H)**. No specific staining was observed using the Santa Cruz sc-22001 antibody **(I-L)**. Dentate gyrus (DG). Scale bar 50 μm.

A thorough validation of two commonly used commercially available Ngb antibodies is presented in Figure 4. On Ngb +/+ the Ngb 4836/5 antibody gave scarce staining in the cortex (Figure 4A) and no staining in the hippocampus (Figure 4A). Strong staining could be seen in the laterodorsal tegmental nucleus (LDTg) (Figure 4C). No immunostaining was seen in Ngb -/- mice (Figure 4B, D). The N7162 antibody from Sigma-Aldrich stained strongly neurons in the hippocampus, cortex and LDTg (Figure 4E, G). In Ngb -/- mice only the staining in the LDTg was abolished. Using the goat sc-22001 antibody only unspecific staining was observed (Figure 4I-L). The Ngb antibodies from Sigma-Aldrich and Santa Cruz tested here can therefore be considered unreliable for IHC characterization of Ngb expression in the mouse brain.

## Discussion

Several studies have investigated the potential neuroprotective effect of Ngb in brain ischemia [8]. Ischemic rodent models have shown conflicting results ranging from no differential expression and no neuroprotective properties [38] to an increase in ischemic infarct size after viral down regulation of Ngb *in vivo*[12], and up-regulation of Ngb expression in WT mice after an ischemic insult [9]. Furthermore, Ngb over-expressing transgenic mice exhibited smaller infarct volumes [10] and reduced levels of oxidative stress markers in the brain after transient focal [6] and global [11] ischemia. There is therefore still no clear picture of how or if Ngb has a role in neuroprotection.

In the current study, we refined the experimental design; (1) using a Ngb-null mouse model; (2) investigating the potential neuroprotective role after pMCAO under strict control of physiological parameters; (3) using design-based stereology for infarct volume estimations.

### Model considerations

#### Analysis of Ngb-null mice has advantages compared to other model systems

Abnormalities may have been inferred if Ngb was essential for sustaining neuronal viability; however Ngb-null mice showed no phenotypic alterations in growth, bodyweight, or overt appearance and behavior when compared to WT littermates. Lack of Ngb did not affect postoperative survival, indicating that loss of Ngb function does not have significant vital impact under conditions with focal brain ischemia. Viral down regulation has been used to study the impact of subnormal Ngb expression levels on brain infarct volumes [12]. The present study avoided the uncertainties associated with the efficiency and possible side effects of viral gene transfer by using genetically Ngb-deficient mice to test whether lack of Ngb degrades the outcome of ischemic brain injury. Still we cannot exclude embryonic Ngb deficiency may have allowed for compensatory mechanisms to mask the Ngb-null phenotype, which is not the case in the viral down-regulation model. The use of a ubiquitous promoter for driving Ngb up-regulation in transgenic models results in Ngb expression in all neurons, including neurons, which do not express Ngb protein naturally, and therefore, represents a non-physiological state. Based on these considerations the use of Ngb-null mice represents a more physiological relevant model when investigating potential endogenous neuroprotective properties of Ngb.

#### Pros and cons of the ischemia mouse-models

In distal permanent MCAo models the artery is occluded distal to the lenticulostriate arteries, hence blood flow to the basal ganglia is not blocked. Ischemic damage is therefore purely cortical [15]. Distal MCAo was selected because damage affects the same cortical area as the transient MCAo (tMCAo) model, but pharmacologic salvage of the penumbra mainly is achievable in cortex [39]. Hence distal MCAo is the preferable model to test for infarct reducing properties of a potentially neuroprotective agent [39]. Most importantly, the method is known for its very high reproducibility in the produced infarct volumes compared to tMCAo [14]. Strict control and monitoring of physiological parameters was part of our experimental set-up to control for possible confounding factors causing variation in infarct volumes [27]. These critical factors include: 1. body temperature; 2. ventilation and blood gas values; and 3. blood pressure. The aforementioned factors have been reported to influence stroke model outcome tremendously [27] and have unfortunately not been reported in all studies where Ngb is postulated to be neuroprotective. Although the animals were operated in randomized order the majority of Ngb-null operations took place in the beginning of the experiment. We believe the difference in anesthesia time is due to the researcher becoming more experienced in carrying out the operations causing a drop in anesthesia time. Sevoflurane is known to be mildly neuroprotective in cerebral ischemia it can therefore not be excluded that the difference in anesthesia time has affected the infarct voume. However the perfect anesthetic with absolutely no influence on neuronal survival is yet to be found and so far sevoflurane is the less neuroprotective one available.

#### Behavioral testing

In the rat there are several well-established behavioral tests that show deficits for weeks after ischemic damage [40-42]. Much less is described regarding sensorimotor deficits in mice. In one of the more extensive studies in mice researchers demonstrated deficits on a large number of behaviors [43], however they used CD1 mice. Also the adhesive removal test has been shown to be sensitive in mice, but here they used Swiss mice [32]. Clear differences exists in the behavioral deficits seen in rat vs. those seen in mice after stroke [43]. Therefore extrapolation of rat behavioral tests to mouse behavior is likely to be misleading. Mice also tend to have increased spontaneous locomotion compared to larger rodents, making sensorimotor tasks more difficult to evaluate. At the same time strain differences in performance on many of the cognitive and sensorimotor tasks have been identified in mice [44-49] thus making it very problematic to choose which behavioral studies to conduct in a given experiment. Also several problems with using transgenic knock-out animals have been identified [50-52]. Inactivating a gene may induce morphological or physiological abnormalities that can complicate interpretation of discrete behavioral effects. Also unexpected compensatory mechanisms might be activated when a gene is missing. Although C57Bl6 mice are known to be a more hyperactive and easily stressed strain compared to both CD1 and Swiss mice, behavioral tests are quite standard in C57Bl6 mice after ischemia [53]. It therefore seems our in house breed sub strain is more aggressive than the average C57Bl6 mouse.

These above mentioned problems might be some of the reasons as to why we have inconclusive results compared to other researchers who have used other strains/substrains of mice in their behavioral studies.

Also the size of the severity of the infarction has to have reached a critical threshold to be detectable [54]. In case of proximal MCAo, behavioral deficits are readily observable in rats [55,56] and mice [57,58] through several classic sensorimotor tests. However as earlier descriebed, proximal MCAo is not representative of all clinical situations because it leads to brain infarctions that are relatively larger than those often observed in human stroke (for review see [59]. Conversely, distal MCAo is more relevant to those clinical situations as it induces smaller infarcts [14,60]. Behavioral alterations after distal MCAo have been largely explored in rat, but, unfortunately only motor coordination difficulty, possible attention deficits and a low increase in eye movement during the dark phase of sleep have been reported in mice [61-63]. The reason for this lack in literature is that behavioral deficits are difficult to detect in mice. Iadecola et al [64] explained that they had to proximally occlude the artery “because distal MCA occlusion produced no neurological deficits” in the mouse. We also performed the distal MCAo and therefore we did not expect to be able to detect neurological deficits. At the same time a close correlation between histology and behavioral outcome is an exception rather than the rule. For review see [65].

Due to the above mentioned reasons we do not believe that functional testing would be sensitive enough to measure the degree of stroke injury or detect any neuroprotective effect of NGB after pMCAo.

### Effect of ischemia on Neuroglobin expression and antibody validation

Studies have reported that ischemic injury leads to an increase in the expression of Ngb in the surrounding penumbra area [9,12,66]. In accordance with Hundahl et al 2006 [38] we observed no increase in Ngb expression within or adjacent to the penumbra zone. Nor did we detect any selective sparing of Ngb expressing neurons (Figure 2B). Western blot analysis of Ngb protein expression in the WT pMCAo versus uninjured sham operated mice revealed a significant down-regulation of Ngb and thus further substantiating that Ngb protein expression not seems to be up-regulated in the pMCAo ischemia model used in this study.

The substantial differences in Ngb expression reported in different studies are probably due to the fact that different antibodies were used. Differences in antibody specificity is a common problem [67]. The Ngb antibodies used in this study as well as in our previous work [33] is confirmed by in-situ-hybridization (ISH) investigations. The Ngb expression pattern reported by us also matches the previous ISH pattern reported by Mammen et al 2002 [68] and the Allen Brain Atlas (http://www.brain-map.org). Furthermore, our Ngb antibodies produce no staining when applied to Ngb-null mice [13,69]. Differences in antibody specificity may therefore be one reason why there has been inconsistencies regarding Ngb expression after ischemia.

#### Infarct volume

In this study infarct volumes were estimated using design-based stereological tools. The advantage of this approach is that information about three-dimensional, microscopic structures may be obtained from thin tissue sections [70]. Unexpectedly, WT mice developed significantly larger infarct volumes after pMCAo when compared to Ngb-null mice (Figure 3). This result is contradictory to the results presented by Sun et al 2003 [12] who found viral down regulation of Ngb to increase infarct volume in cerebral cortex significantly. This difference may arise from several confounding factors such as the way Ngb was deleted/down-regulated, choice of species and ischemia model. In the present study the Ngb-null status was germ-line transmitted and Ngb was at no time present in these mice whereas in the study by Sun et al. adenovirus was used to induce Ngb down-regulation in adult rats and two different ischemia models were used. It is well known that embryonic deletion of a gene may evoke compensatory mechanisms, which are difficult to account for. In Ngb-null mice we observed a differential regulation of glycolytic pathways and Hif1α when exposed to hypoxia [13]. Preconditioning mice with hypoxia and regulation of the aforementioned pathways have been shown to decrease the ischemic brain infarct (for a review see [71,72]), and may thus account for the observed reduction in ischemic infarct size seen in the present study. Similarly, Ngb has been proposed to act as a oxygen sensor in the brain (for review see [73]) and blockage of oxygen sensors using iron chelators like desferrioxamine and CoCl_2_ results in a significant reduction in ischemic brain injury similar to what is seen with hypoxia preconditioning [74-76]. It most be noted that the effect of Ngb deficiency on infarct size following long-term ischemia remains to be investigated and it can therefore not be excluded that a different outcome may occur in a more chronic ischemic state.

Taking the Ngb expression pattern in to account the reduction of cortical infarct size in Ngb-deficient mice found in this study may seem unlikely when considering the sparse cortical expression pattern of Ngb [13]. However, in Ngb-null mice, hypoxic stress induces a global increase in the expression of c-Fos-immunoactivity [13]. This implies that Ngb deficiency may influence the brain in a manner not limited by the anatomical distribution of Ngb. More importantly, however, the significantly smaller infarct size associated with Ngb-deficiency in this experiment argues against the notion of Ngb as a neuroprotective agent.

## Conclusion

This study raises doubt concerning the notion of Ngb being potentially protective for neurons following ischemic injury *in vivo* when expressed at endogenous levels. In conjunction with the present study we recently found that Ngb-deficiency appears to enhance the expression of a few hypoxia-dependent response genes such as Hif1α and c-Fos [13]. Also hypoxia-regulated Ngb seem to contribute to cellular adaptation to hypoxia through a NO mediated pathway [77]. We thus hypothesize that Ngb at endogenous levels may play a role in oxygen sensing in the brain. This could precondition the Ngb-null mice and result in less susceptibility to hypoxia or ischemia compared to WT mice. Further investigations are needed to test this hypothesis.

## Competing interests

The authors declare that they have no competing interests.

## Authors' contributions

CAH, JK and AHS – initiated and planed the study. ZR – executed and planed the pMCAo operations and behavioral tests. ZR – made the stereological measurements. JRN – supervised the stereological measurements. ZR – made the statistical analysis. ZR and CAH – executed the immunohistochemistry and western blotting. ZR – wrote the manuscript. CAH, JK and AHS – critically modified the manuscript. All authors read and approved the final version of the manuscript.
