# Correction: Reduced infarct size in neuroglobin-null mice after experimental stroke *in vivo*

**DOI:** 10.1186/2040-7378-5-10

**Published:** 2013-08-08

**Authors:** Zindy Raida, Christian Ansgar Hundahl, Jesper Kelsen, Jens Randel Nyengaard, Anders Hay-Schmidt

**Affiliations:** 1 Department of Neuroscience and Pharmacology, Faculty of Health Sciences, University of Copenhagen, Copenhagen, Denmark; 2 Department of Physiology, University of Tartu, Tartu, Estonia; 3 Centre of Excellence for Translational Medicine, University of Tartu, Tartu, Estonia; 4 Department of Clinical Biochemistry, University Hospital Bispebjerg, Copenhagen, Denmark; 5 Department of Neurosurgery, University Hospital Copenhagen (Rigshospitalet), Copenhagen, Denmark; 6 Stereology and Electron Microscopy Research Laboratory, Centre for Stochastic Geometry and Advanced Bioimaging, Aarhus University, Aarhus, Denmark; 7 The Panum Institute; Department of Neuroscience and Pharmacology, University of Copenhagen, Blegdamsvej 3, 2200, Copenhagen N, Denmark

## Correction

Since the publication of our article [1] it has come to our attention that our Discussion inadvertently includes some sentences taken from the following articles by DeVries *et al.*[2] and Bouet *et al*. [3] without appropriate citation, using quotation marks:

DeVries AC, Nelson RJ, Traystman RJ, Hurn PD: **Cognitive and behavioural assessment in experimental stroke research: will it prove useful?***Neurosci Biobehav Rev* 2001, **25**:325–342.

Bouet V, Boulouard M, Toutain J, Divoux D, Bernaudin M, Schumann-Bard P, Freret T: **The adhesive removal test: a sensitive method to assess sensorimotor deficits in mice.***Nat Protoc* 2009, **4**:1560–1564.

The authors apologize sincerely for this oversight.
